# Early Detection of SARS-CoV-2 Epidemic Waves: Lessons from the Syndromic Surveillance in Lombardy, Italy

**DOI:** 10.3390/ijerph191912375

**Published:** 2022-09-28

**Authors:** Giorgio Bagarella, Mauro Maistrello, Maddalena Minoja, Olivia Leoni, Francesco Bortolan, Danilo Cereda, Giovanni Corrao

**Affiliations:** 1 Directorate General for Health, Lombardy Region, 20124 Milan, Italy; 2 Agency for Health Protection of the Metropolitan Area of Milan, Lombardy Region, 20122 Milan, Italy; 3 Local Health Unit of Melegnano and Martesana, 20070 Milan, Italy; 4 Unit of Biostatistics, Epidemiology and Public Health, Department of Statistics and Quantitative Methods, University of Milano-Bicocca, 20126 Milan, Italy; 5 National Centre for Healthcare Research and Pharmacoepidemiology, University of Milano-Bicocca, 20126 Milan, Italy

**Keywords:** syndromic surveillance, SARS-CoV-2, early detection, emergency department, EWMA models

## Abstract

We evaluated the performance of the exponentially weighted moving average (EWMA) model for comparing two families of predictors (i.e., structured and unstructured data from visits to the emergency department (ED)) for the early detection of SARS-CoV-2 epidemic waves. The study included data from 1,282,100 ED visits between 1 January 2011 and 9 December 2021 to a local health unit in Lombardy, Italy. A regression model with an autoregressive integrated moving average (ARIMA) error term was fitted. EWMA residual charts were then plotted to detect outliers in the frequency of the daily ED visits made due to the presence of a respiratory syndrome (based on coded diagnoses) or respiratory symptoms (based on free text data). Alarm signals were compared with the number of confirmed SARS-CoV-2 infections. Overall, 150,300 ED visits were encoded as relating to respiratory syndromes and 87,696 to respiratory symptoms. Four strong alarm signals were detected in March and November 2020 and 2021, coinciding with the onset of the pandemic waves. Alarm signals generated for the respiratory symptoms preceded the occurrence of the first and last pandemic waves. We concluded that the EWMA model is a promising tool for predicting pandemic wave onset.

## 1. Introduction

Over the centuries, humanity has experienced multiple types of disasters [1,2], among which pandemics have had a significant role [3]. Recent studies show that new pandemics increased in the last century [4]. Some of these pandemics exert a global influence such as the COVID-19 one, which, as of 18 September 2022, had killed 6.5 million individuals and infected approximately 611 million individuals [5]. As a result, the need for policymakers to be able to control the spread of a pandemic [6] is becoming more relevant by the day. The extensive task of developing increasingly sophisticated mathematical models able to consider the complex dynamics of the pandemic spread has been outlined, including the influence of socioeconomic factors [7] and multi-strain pathogens [8,9,10].

An emerging approach that uses epidemiologic modelling to support policymakers in anticipating the emergence of new epidemics or waves of ongoing epidemics/pandemics is the so-called syndromic surveillance. The latter has been defined as the ongoing systematic collection, analysis, and interpretation of ‘syndrome’-specific data for the early detection of public health aberrations [11]. Syndromic surveillance systems seek to use existing health data in real-time to provide immediate guidance for policymakers [12].

Statistical detection algorithms are critical in any syndromic surveillance system [13]. One of the most popular methods used for the early detection of a respiratory syndrome outbreak is the residual chart model known as the cumulative sum (CUSUM) [14,15]. Similar methods received less attention from public health agencies [16], although some of them (e.g., the exponentially weighted moving average (EWMA) chart) are characterized by exciting properties and greater flexibility compared to the CUSUM residual chart [17,18].

Data used to feed the statistical detection algorithms should be carefully considered, as they strongly affect system performance. Specifically, candidate predictors to detect the onset of an epidemic outbreak early and reliably may conventionally use structured data and consolidated flows (e.g., coded diagnoses of respiratory syndromes recorded in emergency department [ED] logs [19]), as well as unstructured data (e.g., free textual data reported in ED admissions records [20]).

In this study, we used the widely available data from the first 21-month period of the COVID-19 (the disease caused by SARS-CoV-2 infection) pandemic to implement the EWMA chart and compare the performance of two predictor families (i.e., routinely collected structured and unstructured ED data, based on the ICD-9-CM codes and free text description of symptoms, respectively) for early identification of the appearance of the first cases of SARS-CoV-2 infection, as well as the subsequent epidemic waves, that had occurred in an area of Lombardy (Melegnano, Italy), near the municipality where the first European cases of COVID-19 were identified (Lodi, Italy; 27 February 2020). This study was conducted with the help and support of the Lombardy Health Authority as a training test to develop and implement the regional syndromic surveillance system.

## 2. Materials and Methods

### 2.1. Setting

This study used records from three EDs of a local health assessment unit in Lombardy (ASST of Melegnano and Martesana), Italy, which is situated south of the metropolitan area of Milan and covers 53 municipalities and ~630,000 inhabitants (i.e., the target population). Between 1 January 2011 and 9 December 2021, 1,282,100 ED visits were recorded, of which 139,474 took place during the SARS-CoV-2 outbreak, averaging 383.9 or 212.6 visits per day during the pre-epidemic (between 1 January 2011 and 23 February 2020) and epidemic (between 24 February 2020 and 9 December 2021) periods, respectively. During the epidemic, 59,611 nasopharyngeal swabs (which were processed by a Regional Health Authority-accredited laboratory) were positive for SARS-CoV-2.

### 2.2. Data Mining

In this study, we were interested in monitoring the daily number of ED visits relating to respiratory syndrome; two methods were used to classify these visits. The first method encoded patient records using the ICD-9-CM (International Classification of Diseases, 9th Revision, Clinical Modification Reference) code. The respiratory syndrome ICD-9-CM codes used in this study are listed in the Supplementary Material S1. The second method involved the inspection of an individual’s medical records following an ED visit. Medical records contain a free-text section in which the physician writes down the main symptoms detected during the admissions visit. The content of the free text was processed using text mining tools available in a specific package of the Statistical Analysis System [21]. Briefly, text mining tasks were tokenized (which involves splitting the document up into sections called tokens), filtered (to remove unnecessary parts of text such as punctuation), lemmatized (to group inflected forms together into a single base form that could be analyzed as a single entity), and derived (to generate a form, theme, or word) from a pre-existing root or word. The frequency distribution of the words and tokens was then obtained, and the most used terms corresponding to respiratory system symptoms were selected. Overall, 88,036 individual words were identified, resulting in 8,470,168 terms. The words most frequently used to describe respiratory symptoms were “dyspnoea”, “cough”, and “respiratory”. These three terms were collectively combined into a single group, which we will refer to as “respiratory symptoms” from now on.

### 2.3. Statistical Methods

#### 2.3.1. Statistical Process Control (SPC)

Statistical process control (SPC) procedures are based on the idea that even if a process remains the same, observations of that process will exhibit “natural” variability in a statistical sense [22]. For instance, although there might be a “natural” variability in the daily number of ED visits in the respiratory syndrome category, control limits can be set so that most new observations fall within these control limits if the process does not change (i.e., remains in control). However, when the process characteristics do change, the features of the observations also vary, which would result in more observations exceeding the control limits. Consequently, the monitored process might no longer fall within the in-control distribution/range, and the process should be flagged as out of control. This loss of control may occur when an epidemic wave arises due to the persistence of an airborne microorganism in the target population, generating an out-of-control number of ED respiratory syndrome visits. To summarize, exceeding the control limits increases the likelihood that the data-generating process has changed; this process is then considered to be out of control. Distinct steps are required for developing the SPC procedure.

#### 2.3.2. Autoregressive Moving Average (ARMA)

The “natural” variability of the control data is captured and used to establish the in-control distribution. In our study, the time process of interest formed a time series with seasonal variations, meaning that a regression model with an autoregressive moving average (ARMA) with (1, 1) error terms was required to fit the data during the control period preceding the onset of the pandemic in our geographical location (i.e., between 2011 and 2019). A double square root transformation of the daily number of ED visits was made to obtain normality $w_{t}=\sqrt[{4}]{y_{t}}$ where $w_{t}$ is the transformed response variable and $y_{t}$ denotes the number of respiratory syndrome ED visits at day t. The standard format of the regression model was:

$$w_{t}=\mu_{w_{t}}+\varepsilon_{t\;}\;t=1,2,\ldots$$

where $\mu_{w_{t}}$ is the mean response, which depended on a set of predictor variables (e.g., the month of the year, the day of the week, holidays, or trend effects), and $\varepsilon_{t}$ is an error term that follows an ARMA (1, 1) process:

$$\epsilon_{t}=\phi_{1}\varepsilon_{t-1}+\beta_{t\;}-\theta_{1}\beta_{t-1\;}$$

where $\phi_{1}$ and $\theta_{1}$ are the AR (autoregressive) and MA (moving average) coefficients, respectively.

The predictors’ effects were estimated for the following dummy variables: (i) Month of the year: M1 to M11 stand for January to December, with July as a reference; (ii) day of the week: D1 to D6 stand for Monday to Sunday, with Wednesday as a reference; and (iii) holidays: C1, C2, C3, etc., refer to the holiday period and a day either side of this period, with other days as a reference. The sine and cosine functions were used to allow for seasonal effects. Finally, we included the trend variable t. In summary, the mean response was modelled as:

$$\mu_{W_{t}}=\beta_{0}+\overset{11}{\underset{i=1}{\sum }}\beta_{i}M_{i}+\overset{6}{\underset{i=12}{\sum }}\beta_{i}D_{i}+\overset{3}{\underset{i=18}{\sum }}\beta_{i}C_{i}+\beta_{21}sin\;\left(\frac{2\pi t}{365.25}\right)+\beta_{22}cos\;\left(\frac{2\pi t}{365.25}\right)+\beta_{23}t$$

#### 2.3.3. The Exponentially Weighted Moving Average (EWMA) Chart

When monitoring starts, and incoming data are compared with the in-control distribution, to determine if and when the process goes out of control, monitoring is commonly visualized as a control chart, whereby process scores are plotted against time. The EWMA procedure, introduced by Roberts in 1959 [23] to detect mean changes over time, was used in the present study. EWMA combines past and current information and tracks a weighted sum of the original observations, whereby the most recent observations receive higher weights [24]. At each measurement time point within the monitoring period (i.e., each day between 1 January 2020 and 31 December 2021, with *i* = 1, …, *t*), the exponentially weighted moving average *z_i_* was calculated using the following formula:

$$Z_{t}=\lambda \;x_{t}+\left(1-\lambda \right)\;z_{t-1}$$

where *x_t_* denotes the observation at measurement time point *t* and the starting value *z*_0_ is equal to the first step average $\hat{\mu }$_1_. The parameter 0 < *λ* ≤ 1 is the weight given to the current observation and thus also determines the rate at which the weights of the past observations decrease. Although it has been suggested that *λ* values between 0.05 and 0.10 work well when using daily averages [25], we empirically assessed the *λ* value, thus maximizing the performance of the model in our application.

The control limits and central line of the EWMA chart are calculated using the formula below, where *μ*_0_ is the centerline [26].

$$\mu_{0}\pm L\sigma \sqrt{\left(\frac{\lambda }{2-\lambda }\right)\left[1-{\left(1-\lambda \right)}^{2t}\right]}$$

In this way, the EWMA chart was generated for detecting potential “alarm” signals (i.e., better outlier signals). An outlier is defined here as any individual observation that falls outside the control limits established by the EWMA control chart. A decision-making rule was adopted by considering a relevant sequence of at least seven consecutive daily out-of-control signals, calling the latter sequence the “prolonged outlier” hereafter.

Data from SARS-CoV-2-positive nasopharyngeal swabs of the target population (i.e., the official measure of epidemic spread provided by laboratories accredited by the Regional Health Authority) were collected and compared with the EWMA chart results (i.e., the prolonged outlier generated by the process described earlier).

### 2.4. Model Performance

The timeliness of detection was assessed qualitatively by plotting the simulated outbreak signal curves, as well as the occurrence of modelled single and prolonged outliers, against the density of alarms generated per day. In addition, we measured the process performance as follows. Thresholds ranging from 100 to 500 new positive swabs per day, beyond which the spread of infections was considered alarming, were defined. These thresholds corresponded to incidence rates ranging from 16 to 80 cases per 100,000 person-days and were calculated by reviewing a wide range of alert thresholds from several countries [27,28]. To mitigate random fluctuations, the data were revised by calculating moving averages centered at seven days. Each day of the monitoring period was inspected, and a prolonged outlier signal (from the control card) was regarded as a true positive if the incidence threshold (from the nasopharyngeal swabs) was overcome on that day or within seven days. Conversely, a prolonged outlier signal was regarded as a false positive if the incidence threshold was not overcome either on that day or within seven days. The sensitivity (i.e., the number of days that exceeded the threshold for defining a true epidemic outbreak and were labelled as true positives by the control card) was plotted against 1–specificity (i.e., the number of days that did not exceed the threshold and were labelled as false positives by the control card) to show the ability of the control card to detect outbreak onset.

Readers interested in replicating the results of this paper can write to the corresponding author (GC) to receive the code and data used as part of this research.

## 3. Results

**Figure 1:**
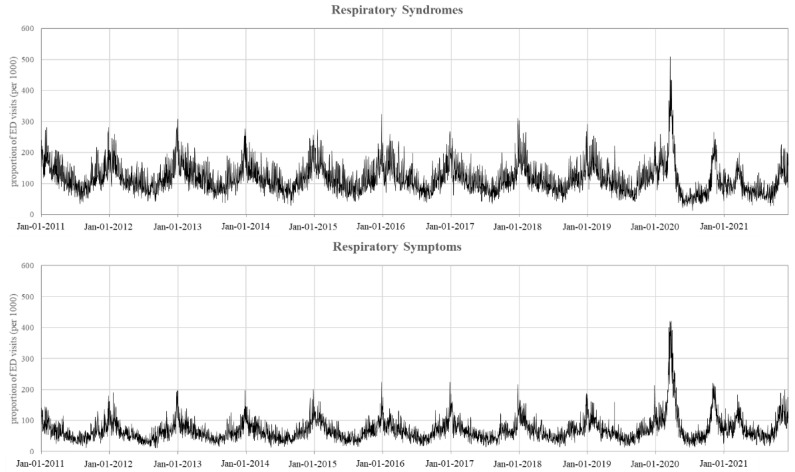
The observed daily number of ED visits associated with either respiratory syndrome read codes or recognized through respiratory symptoms extracted from the free text of medical records, between 1 January 2011 and 9 December 2021.

During the study period, 150,300 ED visits were encoded as being associated with a respiratory syndrome (i.e., from structured coded data), while for 87,696 ED visits, respiratory symptoms were recognized via the text mining process (i.e., from unstructured free text data); the corresponding average proportions were 11.7% and 6.8%, respectively. The daily number of ED visits detected using either respiratory syndrome read codes or through text mining for respiratory symptoms are shown in Figure 1. We evaluated data from the 2011–2020 period and found that the peak of respiratory syndromes and symptoms occurred between the months of December and January. Four COVID-19 pandemic waves, characterized by an increase in the number of respiratory ED visits, occurred between March 2020 and November 2021. It should be noted that (i) the first wave (March 2020) was characterized by more than half of the ED visits having a respiratory syndrome read code (compared to values of approximately 30% for the other peaks); (ii) similarly, in March 2020, approximately 40% of the ED visits were recognized as being associated with respiratory symptoms, while during the other periods these symptoms comprised, at most, 20% of the visits; and (iii) the conventional December-January peak did not occur between 2020 and 2021. Parameters of the regression model with ARMA (1, 1) errors (Supplementary Material S2) clearly show a systematic component of time series data (Figure 1).

As expected, the peak period for the respiratory syndromes and symptoms occurred between December and January of each year, and during the holidays and adjacent weekdays. A decreasing yearly trend for both the considered outcomes was also observed.

**Figure 2:**
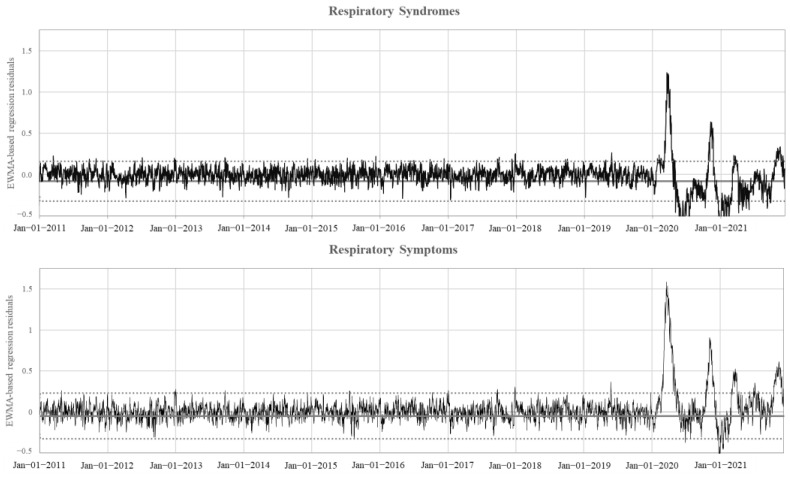
Model-based residual of the daily trends in the number of ED visits with either respiratory syndrome read codes or recognized through respiratory symptoms recorded in the free text of medical records, between 1 January 2011 and 9 December 2021. Mean value of the entire series and the upper/lower control limits used to prepare the exponentially weighted moving average (EWMA) control chart are shown.

Figure 2 shows the model-based residual of the daily trends in the proportion of ED visits recognized as being associated with respiratory syndromes and symptoms, displayed as EWMA charts. Few and modest signals appeared in the period between 2011 and 2019, while four very intensive outliers of respiratory syndromes and symptoms occurred in March and November 2020 and 2021. A further peak of respiratory symptoms was observed in July 2021. It should be noted that the peaks were systematically more intense for symptoms than for syndromes.

**Figure 3:**
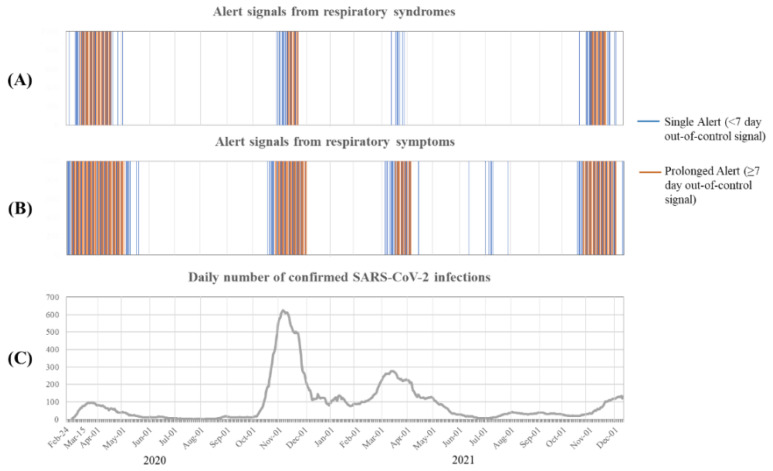
Comparing single and prolonged outlier daily signals generated from respiratory syndromes (**A**) and symptoms (**B**) and daily number of confirmed SARS-CoV-2 infections (**C**) during the period from 24 February 2020 to December 2021. Daily outlier signals were obtained by the Exponentially Weighted Moving Average control chart; colored vertical lines indicate days when the signal occurred; it was blue when only a single Alert (<7 day out-of-control signal) occurred, and red when the signal also regarded a prolonged Alert (≥7 day out-of-control signal). Seven-day mobile average was used for representing the daily number of confirmed SARS-CoV-2 infections.

Figure 3 shows a comparison of EWMA charts. Specifically, the individual and prolonged outliers generated during the period between 2 February 2020 and 9 December 2021 were compared to the daily number of positive SARS-CoV-2 cases during the same period. As expected, very few infections were recorded at the start of the pandemic because the sudden shock of infection by a previously unknown agent had not allowed for adequate tracking of cases. Thus, only a small proportion of the population was monitored for SARS-CoV-2 infection. We found that the alarms generated from the monitoring of respiratory syndromes, and even more so from the monitoring of respiratory symptoms, always coincided with the confirmed rate of SARS-CoV-2 infection. In some cases, outliers preceded the appearance of the peak detected by a positive nasopharyngeal swab. This was particularly true for the first and last epidemic waves. Furthermore, the monitoring of respiratory symptoms was a better predictor than the monitoring of respiratory syndrome code lists.

**Figure 4:**
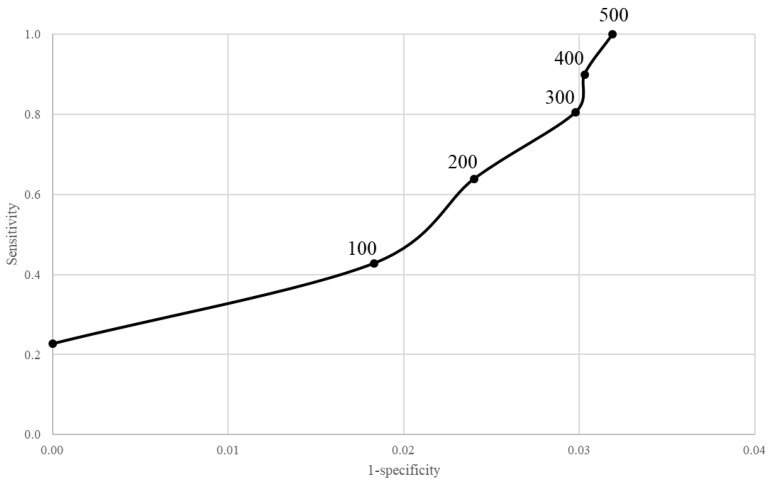
A plot of sensitivity against 1-specificity of the EWMA control chart of respiratory syndromes and symptoms for detecting the outbreak onset by varying the threshold of the true outbreak. Thresholds for the number of positive COVID-19 cases, above which the transmission of SARS-CoV-2 became alarming, are reported in the figure for each combination of sensitivity and 1-specificity.

Figure 4 shows plots of sensitivity versus 1-specificity of the respiratory symptoms EWMA chart to predict SARS-CoV-2 outbreaks under varying thresholds of detection. As shown in Supplementary Material S3, the best fitting model used a *λ* value of 0.4. Virtually all the alarms generated by the incidence of new cases (≥500 positive swabs per day, corresponding to 160 cases per 100,000 person-days) were identified by the control card, with a false positive rate of 3.3%. On the other hand, by reducing the threshold to 300 cases per day, the EWMA control chart had a lower sensitivity (80%), while only a slight improvement in the false positive rate was observed (3.0%).

## 4. Discussion

Our results suggest that the careful analysis of ED visit data for the presence of respiratory syndromes or symptoms is useful for detecting the start of respiratory syndrome outbreaks, and particular those that develop rapidly. From the comparison of candidate predictors, we found that alarm signals obtained from the mining of free text from medical reports were more effective at predicting pandemic onset than those generated using ICD-9-CM codes. Finally, we did not observe that the analysis of ED visits led to the early prediction of respiratory outbreaks in pandemic waves that followed the first wave. This is likely due to the fact that these later epidemics were caused by a variant very different from the original one, as was the case of the omicron variant of the fourth epidemic wave [29].

Some additional issues deserve to be addressed. First, CUSUM chart models have been extensively used for syndromic surveillance systems worldwide [13,14,15]. Although we did not attempt a between-model comparison, we observed that the residual EWMA chart worked well in showing abnormal increases in daily counts of respiratory-syndrome visits. Thus, this model could potentially be used to support empirical evidence gained in other fields [16,30,31,32], beyond that of the influenza epidemic [33,34]. This is not surprising because, as EWMA calculates a moving average of current and past observations, it is resistant to deviation from normality and thus useful for plotting individual values [35,36]. Furthermore, although extensive comparisons revealed that EWMA control schemes have similar average run length properties to those of CUSUM control schemes, EWMA was particularly well suited to detecting small shifts in the process mean [37]. On the other hand, the EWMA chart had been found to be more prone to false alarm counts with respect to other approaches [38], and this potential weakness should be careful considered in a surveillance syndromic system.

Second, we used official data on the positivity of nasopharyngeal swabs as a proxy for the gold standard, that is, the daily count of SARS-CoV-2 infections. However, it should be considered that the proxy systematically underestimates the gold standard and that this level of underestimation changes over time. For example, only a small proportion of infected individuals was detected both at the beginning of the epidemic (a period during which the population was not prepared for the emergency) and during the fourth epidemic wave (when most infections occurred with no or few symptoms). It is noteworthy that the syndromic surveillance system we proposed worked better on the latter two occasions, that is, when the conventional tracking system fails for a yet-to-be-determined reason. On the other hand, with respect to conventional surveillance systems, the syndromic system based on respiratory symptom data may lack generalizability as symptom description is directly influenced by the awareness of patients as well as the diagnostic behavior of physicians.

## 5. Conclusions

Although syndromic surveillance systems for influenza-like illnesses are primarily designed for informing situational awareness rather than actual case finding in an epidemic setting [39], our results suggest that large-scale surveillance networks in the community could be useful as a population-based tool. In this setting, the system would serve to enhance our understanding of the full spectrum of disease, especially in the early phase of an evolving epidemic [40]. Importantly, future work should integrate data from cases detected by syndromic surveillance systems with data from other sources (e.g., the telehealth service, internet searches, laboratory testing, and wastewater detection).

In conclusion, our findings suggest that a syndromic surveillance system using EWMA chart models to process the free text of ED acceptance records is promising, and its performance deserves to be replicated using other study periods and data from different countries. Because the COVID-19 pandemic can be considered the first major pandemic of the 21st century, more empirical studies are needed to help health systems prepare for future pandemics. Meanwhile, enriching the current syndromic surveillance systems with other and unusual proxies is strongly recommended to support policymakers in anticipating the emergence of new epidemics or waves of ongoing epidemics/pandemics.

## Data Availability

The data used in this study were obtained from the Lombardy Region under a licence. Since restrictions apply to the availability of these data, requests should be made directly to the Lombardy Region.

## Supplementary Materials

The following supporting information can be downloaded at: https://www.mdpi.com/article/10.3390/ijerph191912375/s1, S1: List of ICD-9-CM codes used to track respiratory syndromes; S2: Parameters of regression model with ARMA (1, 1) errors; S3: Values of sensitivity and 1-specificity for detecting the outbreak onset for increasing threshold of true outbreak according to different values of the *λ* parameter (see Methods).

## References

[1] Noji E.K., Toole M.J. (1997). The Historical Development of Public Health Responses to Disasters. Disasters.

[2] Van Bavel B.J.P., Curtis D.R. (2016). Better Understanding Disasters by Better Using History. Int. J. Mass Emerg. Disasters.

[3] Conti A.A. (2020). Historical and methodological highlights of quarantine measures: From ancient plague epidemics to current coronavirus disease (COVID-19) pandemic. Acta Biomed..

[4] Lazebnik T., Bunimovich-Mendrazitsky S. (2022). Generic approach for mathematical model of multi-strain pandemics. PLoS ONE.

[5] Our World in Data. Statistics and Research. Coronavirus Pandemic (COVID-19). https://ourworldindata.org/coronavirus.

[6] Lazebnik T., Bunimovich-Mendrazitsky S., Labib S. (2021). Pandemic management by a spatio–temporal mathematical model. Int. J. Nonlinear Sci. Numer. Simul..

[7] Brodeur A., Gray D., Islam A., Bhuiyan S. (2021). A literature review of the economics of COVID-19. J. Econ. Surv..

[8] Khyar O., Allali K. (2020). Global dynamics of a multi-strain SEIR epidemic model with general incidence rates: Application to COVID-19 pandemic. Nonlinear Dyn..

[9] Lazebnik T., Blumrosen G. (2022). Advanced multi-mutation with intervention policies pandemic model. IEEE Access.

[10] Arruda E.F., Das S.S., Dias C.M., Pastore D.H. (2021). Modelling and optimal control of multi strain epidemics, with application to COVID-19. PLoS ONE.

[11] Yan P., Chen H., Zeng D. (2008). Syndromic surveillance systems. Ann. Rev. Inf. Sci. Technol..

[12] Henning K.J. (2004). Overview of syndromic surveillance: What is syndromic surveillance?. Morb. Mortal. Wkly. Rep..

[13] Griffin B.A., Jain A.K., Davies-Cole J., Glymph C., Lum G., Washington S.C., A Stoto M. (2009). Early detection of influenza outbreaks using the DC Department of Health’s syndromic surveillance system. BMC Public Health.

[14] Chen H., Huang C. (2014). The use of CUSUM residual chart to monitor respiratory syndromic data. IIE Trans..

[15] Oud M.A., Almuqrin M. (2021). On the early detecting of the COVID-19 outbreak. J. Infect. Dev. Ctries..

[16] Szarka J.L., Gan L., Woodall W.H. (2011). Comparison of the early aberration reporting system (EARS) W2 methods to an adaptive threshold method. Stat. Med..

[17] Neubauer A.S. (1997). The EWMA control chart: Properties and comparison with other quality-control procedures by computer simulation. Clin. Chem..

[18] Albarracin O.Y.E., Alencar A.P., Ho L.L. (2018). Effect of neglecting autocorrelation in regression EWMA charts for monitoring count time series. Qual. Reliab. Eng. Int..

[19] Lall R., Abdelnabi J., Ngai S., Parton H.B., Saunders K., Sell J., Wahnich A., Weiss D., Mathes R.W. (2017). Advancing the use of emergency department syndromic surveillance data, New York City, 2012–2016. Public Health Rep..

[20] Brunekreef T.E., Otten H.G., van den Bosch S.C., Hoefer I.E., Laar J.M., Limper M., Haitjema S. (2021). Text mining of electronic health records can accurately identify and characterize patients with systemic lupus erythematosus. ACR Open Rheumatol..

[21] Chakraborty G., Pagolu M., Garla S. (2013). Text Mining and Analysis Practical Methods, Examples, and Case Studies Using SAS^®^.

[22] Schat E., Ceulemans E. (2022). The Exponentially Weighted Moving Average Procedure for Detecting Changes in Intensive Longitudinal Data in Psychological Research in Real-Time: A Tutorial Showcasing Potential Applications. Assessment.

[23] Roberts S.W. (1959). Control chart tests based on geometric moving averages. Technometrics.

[24] Montgomery D.C. (2019). Introduction to Statistical Quality Control.

[25] Schat E., Tuerlinckx F., Smit A.C., De Ketelaere B., Ceulemans E. (2021). Detecting mean changes in experience sampling data in real time: A comparison of univariate and multivariate statistical process control methods. Psychol. Methods.

[26] Sengupta S., Mohinuddin S., Arif M. (2021). Spatiotemporal dynamics of temperature and precipitation with reference to COVID-19 pandemic lockdown: Perspective from Indian subcontinent. Environ. Dev. Sustain..

[27] https://www.gov.uk/government/publications/uk-covid-19-alert-level-methodology-an-overview/uk-covid-19-alert-level-methodology-an-overview.

[28] https://preventepidemics.org/wp-content/uploads/2020/05/Annex-2_Example-of-an-alert-level-system_US_FINAL.pdf.

[29] Brandal L.T., MacDonald E., Veneti L., Ravlo T., Lange H., Naseer U., Feruglio S., Bragstad K., Hungnes O., Ødeskaug L. (2021). Outbreak caused by the SARS-CoV-2 Omicron variant in Norway, November to December 2021. Eurosurveillance.

[30] Supharakonsakun Y., Areepong Y., Sukparungsee S. (2020). The performance of a modified EWMA control chart for monitoring autocorrelated PM2.5 and carbon monoxide air pollution data. PeerJ.

[31] Baldewijns G., Luca S., Vanrumste B., Croonenborghs T. (2016). Developing a system that can automatically detect health changes using transfer times of older adults. BMC Med. Res. Methodol..

[32] Eren-Oruklu M., Cinar A., Quinn L. (2010). Hypoglycemia prediction with subject-specific recursive time-series models. J. Diabetes Sci. Technol..

[33] Liu L., Yue J., Lai X., Huang J., Zhang J. (2019). Multivariate nonparametric chart for influenza epidemic monitoring. Sci. Rep..

[34] Steiner S.H., Grant K., Coory M., Kelly H.A. (2010). Detecting the start of an influenza outbreak using exponentially weighted moving average charts. BMC Med. Inform. Decis. Mak..

[35] Carson P.K., Yeh A.B. (2008). Exponentially weighted moving average (EWMA) control charts for monitoring an analytical process. Ind. Eng. Chem. Res..

[36] Lipsitch M., Hayden F.G., Cowling B.J., Leung G.M. (2009). How to maintain surveillance for novel influenza A H1N1 when there are too many cases to count. Lancet.

[37] Lucas J.M., Saccucci M.S. (2012). Exponentially Weighted Moving Average Control Schemes: Properties and Enhancements. Technometrics.

[38] Corominas L., Villez K., Aguado D., Rieger L., Rosén C., Vanrolleghem P.A. (2011). Performance evaluation of fault detection methods for wastewater treatment processes. Biotechnol. Bioeng..

[39] Ip D.K., Liao Q., Wu P., Gao Z., Cao B., Feng L., Xu X., Jiang H., Li M., Bao J. (2013). Detection of mild to moderate influenza A/H7N9 infection by China’s national sentinel surveillance system for influenza-like illness: Case series. BMJ.

[40] TRIPLE S Syndromic Surveillance System in Europe. Guidelines for designing and implementing a syndromic surveillance system. https://webgate.ec.europa.eu/chafea_pdb/assets/files/pdb/20091112/20091112_d08_giss_en_ps.pdf.

