# Supplementary material for: Early Detection of SARS-CoV-2 Epidemic Waves: Lessons from the Syndromic Surveillance in Lombardy, Italy
Source: Int J Environ Res Public Health. 2022 Sep 28;19(19):12375. doi: 10.3390/ijerph191912375 (PMC9565943; doi:10.3390/ijerph191912375)
Supplement: Supplementary file 1 [file ijerph-19-12375-s001.zip › ijerph-1860138-supplementary.pdf]

Supplementary Material

S1. List of ICD-9-CM codes used to track respiratory syndromes.

"00322", "0203", "0204", "0205", "0212", "0221", "0340", "0391", "0521", "0551", "05679", "09889", "1048", "1124", "1140", "1144", "1145", "11505", "1173", "1304", "1363", "38100", "38101", "38102", "38103", "38104", "38105", "38106", "3814", "38150", "38151", "38200", "38201", "38202", "3821", "3822", "3823", "3824", "3829", "460", "4610", "4611", "4612", "4613", "4618", "4619", "462", "463", "46400", "46401", "46410", "46411", "46420", "46421", "46430", "46431", "4644", "46450", "46451", "4650", "46500", "4658", "4659", "4660", "46611", "46619", "470", "4710", "4711", "4718", "4719", "4720", "4721", "4722", "4730", "4731", "4732", "4733", "4738", "4739", "47400", "47401", "47402", "47410", "47411", "47412", "4748", "4749", "475", "4760", "4761", "4770", "4771", "4772", "4778", "4779", "4780", "47811", "47819", "47821", "47822", "47824", "47829", "47830", "47831", "47834", "4784", "4785", "4786", "47874", "47875", "47879", "4788", "4789", "480", "4800", "4801", "4802", "4803", "4808", "4809", "481", "4820", "4821", "4822", "48232", "48239", "48240", "48241", "48242", "48249", "48282", "48283", "48289", "4829", "4830", "4831", "4838", "4845", "4847", "4848", "485", "486", "4870", "4871", "4878", "48881", "48882", "48889", "490", "4910", "4911", "49120", "49122", "4918", "4919", "4928", "49300", "49301", "49302", "49310", "49311", "49312", "49322", "49381", "49382", "49390", "49391", "49392", "4940", "4941", "4950", "4951", "4952", "4953", "4954", "4955", "4956", "4957", "4958", "4959", "500", "501", "502", "503", "504", "505", "5060", "5061", "5062", "5063", "5064", "5069", "5070", "5071", "5078", "5080", "5081", "5082", "5088", "5089", "5100", "5109", "5110", "5111", "51181", "51189", "5119", "5120", "5121", "5122", "51281", "51282", "51283", "51284", "51289", "5130", "5131", "514", "515", "5160", "5161", "5162", "5163", "51630", "51631", "51632", "51633", "51634", "51635", "51636", "51637", "5164", "5165", "51661", "51662", "51663", "51664", "51669", "5168", "5169", "5171", "5178", "5180", "5181", "5182", "5183", "5184", "51851", "51852", "51853", "5187", "51881", "51882", "51883", "51884", "51889", "51900", "51901", "51909", "51911", "51919", "5192", "5193", "5194", "5198", "5199", "53084", "769", "78600", "78609", "78630", "78631", "78639", "78650", "78651", "78652", "78659", "7867", "7869", "7991", "99731", "99739", "99811", "9982", "V4614", “48041"

S2. Parameters of regression model with ARMA (1,1) errors.

	Respiratory diagnosis				Respiratory symptoms			
Parameter	Estimate	SE	t - value	Pr > |t|	Estimate	SE	t - value	Pr > |t|
Intercept	3.22777	0.03706	87.10	<.0001	2.70404	0.04084	66.21	<.0001
January (M1)	-0.07139	0.06575	-1.09	0.2776	-0.0009761	0.07269	-0.01	0.9893
February (M2)	-0.11664	0.06401	-1.82	0.0685	-0.04415	0.07050	-0.63	0.5312
March (M3)	-0.16566	0.05822	-2.85	0.0045	-0.10025	0.06423	-1.56	0.1187
April (M4)	-0.13148	0.04971	-2.65	0.0082	-0.03447	0.05475	-0.63	0.5290
May (M5)	-0.03600	0.03930	-0.92	0.3597	0.01678	0.04317	0.39	0.6975
June (M6)	0.03738	0.02791	1.34	0.1805	0.01254	0.03046	0.41	0.6806
July	0		reference		0		reference	
August (M7)	-0.01583	0.02813	-0.56	0.5736	-0.01418	0.03046	-0.47	0.6416
September (M8)	0.0017233	0.03989	0.04	0.9655	0.02184	0.04368	0.50	0.6171
October (M9)	0.02614	0.04996	0.52	0.6008	0.04204	0.05508	0.76	0.4453
November (M10)	-0.05787	0.05847	-0.99	0.3224	-0.08709	0.06471	-1.35	0.1784
December (M11)	0.01049	0.06389	0.16	0.8695	0.02778	0.07096	0.39	0.6954
Monday (D1)	-0.0073402	0.0092623	-0.79	0.4281	-0.01054	0.01120	-0.94	0.3464
Tuesday (D2)	0.0019362	0.0091195	0.21	0.8319	-0.0022361	0.01111	-0.20	0.8405
Wednesday	0		reference		0		reference	
Thursday (D3)	0.0038024	0.0091198	0.42	0.6768	0.0016915	0.01111	0.15	0.8790
Friday (D4)	0.06491	0.0092673	7.00	<.0001	0.01176	0.01120	1.05	0.2942
Saturday (D5)	0.28445	0.0093368	30.47	<.0001	0.17682	0.01125	15.72	<.0001
Sunday (D6)	0.31987	0.0093347	34.27	<.0001	0.21489	0.01125	19.11	<.0001
Working day	0		reference		0		reference	
Holiday (C1)	0.29813	0.01545	19.30	<.0001	0.23621	0.01825	12.94	<.0001
Pre-holiday (C2)	0.14918	0.01653	9.02	<.0001	0.12162	0.01971	6.17	<.0001
Post-holiday (C3)	0.03252	0.01641	1.98	0.0475	0.05450	0.01956	2.79	0.0054
Sine (sin)	0.14884	0.03242	4.59	<.0001	0.08593	0.03591	2.39	0.0168
Cosine (cos)	0.27682	0.03227	8.58	<.0001	0.30290	0.03567	8.49	<.0001
Trend variable (t)	-0.0000225	6,37E-01	-3.54	0.0004	0.00002219	6,83E-01	3.25	0.0012
MA1,1	0.68531	0.03148	21.77	<.0001	0.75831	0.03289	23.06	<.0001
AR1,1	0.86124	0.02269	37.96	<.0001	0.88205	0.02462	35.83	<.0001

The distribution of the residual is normal with a mean zero and SE 0.18 for symptoms and 0.16 for diagnosis. These residuals a_t are used to construct an EWMA chart for monitoring abnormal increases in the proportion of visits.

S3. Values of sensitivity and 1-specificity for detecting the outbreak onset for increasing threshold of true outbreak according to different values of the λ parameter (see Methods).

λ = 0.1

Threshold [a]	Sensitivity [b]	1 – specificity [c]
100	0.61	0.04
200	0.65	0.05
300	0.83	0.06
400	0.93	0.06
500	1.00	0.06

λ = 0.2

Threshold [a]	Sensitivity [b]	1 – specificity [c]
100	0.52	0.03
200	0.67	0.03
300	0.83	0.04
400	0.93	0.04
500	1.00	0.04

λ = 0.4

Threshold [a]	Sensitivity [b]	1 – specificity [c]
100	0.43	0.02
200	0.64	0.02
300	0.81	0.03
400	0.90	0.03
500	1.00	0.03

λ = 0.6

Threshold [a]	Sensitivity [b]	1 – specificity [c]
100	0.18	0.01
200	0.32	0.02
300	0.62	0.02
400	0.70	0.02
500	0.70	0.02

[a] Thresholds ranging from 100 to 500 new positive swabs in each day beyond the which the spread of infections is to be considered "really" alarming

[b] Sensitivity: proportion of days exceeding the threshold that were labelled as true positives by the control card

[c] 1 – specificity: proportion of days no exceeding the threshold that were labelled as false positives by the control card
